# Failure to detect functional transfer of active K-Ras protein from extracellular vesicles into recipient cells in culture

**DOI:** 10.1371/journal.pone.0203290

**Published:** 2018-09-07

**Authors:** Natalie Luhtala, Tony Hunter

**Affiliations:** Molecular and Cell Biology Laboratory, Salk Institute for Biological Studies, La Jolla, CA, United States of America; Scuola Superiore Sant'Anna, ITALY

## Abstract

Exosomes, extracellular nanovesicles that carry nucleic acids, lipids, and proteins, have been the subject of several studies to assess their ability to transfer functional cargoes to cells. We recently characterized extracellular nanovesicles released from glioblastoma cells that carry active Ras in complex with proteins regulating exosome biogenesis. Here, we investigated whether a functional transfer of Ras from exosomes to other cells can initiate intercellular signaling. We observed that treatment of serum-starved, cultured glioblastoma cells with exogenous glioblastoma exosomes caused a significant increase in cellular viability over time. Moreover, we detected fluorescent signal transfer from lipophilic dye-labeled exogenous glioblastoma exosomes into cultured glioblastoma cells. To probe possible signaling from cell-to-cell, we utilized bimolecular luciferase complementation to examine the ability of K-Ras in exosomes to interact with the Raf-Ras Binding domain (Raf-RBD) expressed in a recipient cell line. Although the K-Ras/Raf-RBD interaction was readily detectable upon co-expression in a single cell line, or following lysis of co-cultured cell lines separately expressing K-Ras and RBD, bearing in mind the limitations of our assay, we were unable to detect the interaction in the intact, co-cultured cell lines or upon treatment of the Raf-RBD-expressing cells with exosomes containing K-Ras. Furthermore, HA-Tag-BFP fused to the K-Ras hypervariable region and CAAX sequence failed to be transferred at significant levels from extracellular vesicles into recipient cells, but remained detectable in the cell supernatants even after 96 hours of culture of naïve cells with extracellular vesicles. We conclude that if transfer of functional K-Ras from extracellular vesicles into the cytoplasm of recipient cells occurs, it must do so at an extremely low efficiency and therefore is unlikely to initiate Ras-ERK MAP kinase pathway signaling. These results suggest that studies claiming functional transfer of protein cargoes from exosomes should be interpreted with caution.

## Introduction

Exosomes are tiny (50-150nm) extracellular vesicles (EVs) implicated in cell-to-cell communication. When compared to intact cells, these vesicles are enriched for membrane-associated signaling and cell communication proteins [1], and have been proposed to alter a number of cellular processes, such as prion protein transmission and neurodegenerative diseases [2], regulation of immune functions [3], tumor angiogenesis [4–16], fibroblast signaling to tumors [17,18], and priming of the metastatic niche [19–26]. In addition to lipids and proteins, these vesicles carry DNA and RNA [13,27–29] and are present in biofluids; hence, research is underway to harness these carriers of cell communication cargoes to identify signatures that could be useful biomarkers in human disease [30–35]. Characterizing a direct mechanism by which exosomes mediate cell-to-cell communication could prove valuable to our understanding of the impact of exosomes on physiological processes and could aid in interpreting circulating biomarker observations as well.

Although research continues to uncover more complexity in this process, the mechanism by which exosomes are released from cells is better characterized than that of exosome uptake [36–39]. Membrane-associated cargoes sort to an endosome, and intralumenal sorting of these endosomal cargoes produces multivesicular bodies (MVBs), which carry cargoes within intralumenal vesicles (ILVs). Fusion of an MVB with the plasma membrane releases the ILVs as exosomes within the extracellular space.

The pathways governing the trafficking of exosomes from the extracellular space are not as well defined; however, current literature supports the uptake of exosomes by cells for functional recycling of exosome cargoes within acceptor cells. Detailed microscopic analyses have implicated several different processes, sometimes with conflicting mechanisms, in the uptake of these EVs; endocytosis [40–42], surfing on filopodia to access endocytic sites [43], macropinocytosis [42,44,45], direct fusion with the plasma membrane [46,47] and phagocytosis [48] have all been described. Functional recycling of exosome cargoes following uptake is indicated by studies of both RNA and protein cargoes. Small RNA and mRNA cargoes within exosomes have been demonstrated to directly affect translation of their specific proteins by exosome-to-cell transfer [13,29,49], and exosome protein cargoes display activity-dependent phenotypes when transferred to acceptor cells [50,51] *in vitro*. Particularly relevant to the study of Ras signaling, exogenous exosomes from colon cancer cells carrying the G13D K-Ras mutant, but not the wild-type K-Ras protein, enhanced three-dimensional growth of rat intestinal epithelial cells [52].

We initially hypothesized that active Ras might be transferred to other cells and could allow a heterogeneous population of cells to directly share membrane signaling factors and respond more effectively to stress. In a previous study, we found that glioblastoma (GBM) brain tumor cells release active Ras in extracellular nanovesicles whose properties are consistent with exosomes [1]. In this work, we observed that lipophilic dye-labeled exosomes from GBM cells transferred dye to the cytoplasm of GBM cells and found that treating serum-starved cells with these exosomes exogenously improved their growth over time. Nevertheless, we failed to observe intercellular transfer of K-Ras using a sensitive bimolecular luciferase complementation (BiLC) assay to probe for intercellular transfer of K-Ras leading to its interaction with the Raf-Ras binding domain (RBD), even though complementation could be observed by either intracellular co-expression of the K-Ras and Raf-RBD constructs or by lysing co-cultured cell lines individually expressing these constructs. Finally, we analyzed an HA-Tag-BFP reporter, which has the K-Ras hypervariable region CAAX sequence fused to its C-terminus, and found that exosomes containing this reporter protein were readily detectable outside of cells in the medium, but failed to significantly transfer from exosomes into cells.

## Materials and methods

### Cell culture and stable cell line generation

005 cells [53] and Nf5310 cells (established from mouse tumors induced by lentiviruses carrying shRNA to NF1, p53, and PTEN, a gift of the Verma lab, unpublished results) were cultured as previously described in N2 medium: DMEM:F12 supplemented with 1X N2, hEGF (20 ng/ml), hFGF-2 (20 ng/ml), heparin (40 μg/ml), and L-glutamine to maintain an undifferentiated state. U87MG cells [54,55] were cultured in DMEM (4.5 g/liter glucose, L-glutamine, and sodium pyruvate) supplemented with 10% FBS. Penicillin/streptomycin (Corning, 1X) and ciprofloxacin (10 μg/ml) antibiotics were added to maintain cultures sterile. All cells were cultured at 37°C in 5% CO_2_.

U87MG cells stably expressing lentiviral K-Ras expression plasmids were selected as a batch for puromycin-resistance using 3 μg/ml puromycin and validating complete eradication of control cells cultured in parallel. Puromycin selection was continuously applied to cultures to ensure retention of expression constructs, and tetracycline was excluded from passaged cultures and added only for experimental analyses to promote maintenance of the transgenes and allow isogenic comparisons of K-Ras mutants.

Stable expression of cLuc-Flag-Raf-RBD was achieved by batch selection for blasticidin resistance using 5 μg/ml blasticidin and validating complete eradication of control cells cultured in parallel.

### Lentiviral packaging, purification and transduction

To produce lentiviral stocks, the lentiviral vectors described in Table 1 were transfected into HEK293T cells, and packaging and purification were conducted by following a 3^rd^ generation VSV.G pseudotyped lentiviral packaging protocol [56] with modifications recommended by the Salk viral vector core. For each 15 cm plate of HEK293T cells, 12.2 μg of lentiviral constructs were added, and 8.1 μg, 3.1 μg, 4.1 μg of pMDL, pRev and VSVG vectors were added, respectively. After 4 hr of incubation at 37°C and 10% CO_2_, medium was replaced with DMEM+ 3% FBS (exosome-depleted as described in “Ultracentrifugation harvesting of EVs” below) + 25 mM HEPES. Conditioned media were collected at 72 hr. Plasmid carry-over was removed by digestion with DNase I, and conditioned medium with lentiviral particles was concentrated by ultracentrifugation at 70,000*xg* for 2 hr to use for transduction (without sucrose purification or titering). Lentiviral particles concentrated from the conditioned medium of one 15 cm plate (resuspended in HBSS and cleared) were used to transduce U87MG cells at 70–80% confluency on one well of a 6-well plate by incubating for 24 hr. Media containing lentiviral particles were then removed, and selection media were added.

**Table 1:** Source of plasmids used in this study.

Plasmid #	Name	Source
pRAR3G	TRE3G PURO	Gift of the lab of Dr. Inder Verma
	CMV-nLuc -linker-HA-GGA-TCC-K-Ras WT	Gift of the lab of Dr. Geoffrey Wahl
	CMV-nLuc -linker-HA-GGA-TCC-K-Ras G12D	Gift of the lab of Dr. Geoffrey Wahl
pLi656	CMV-cLuc-linker-HA-GGA-TCC-K-Ras Y40C	Gift of the lab of Dr. Geoffrey Wahl
pLi789	EF1α-Gly-HA-TagBFP-Ser-Gly-K-Ras-HVR-CAAX	Gift of the lab of Dr. Geoffrey Wahl
13338	GST-Raf-RBD	Addgene [57]
pNL6	TRE3G-nLuc-HA-Kras4B-WT	This study
pNL7	TRE3G-nLuc-HA-Kras4B-G12D	This study
pNL8	TRE3G-nLuc-HA-Kras4B-Y40C	This study
pNL9	TRE3G-cLuc-Flag-Raf-RBD	This study

### Cloning of lentiviral vectors

The sequences for primers used in the cloning and sequencing of the constructs described below are given in Table 2.

**Table 2:** Primers used in cloning and sequencing.

Primer Name	Sequence 5'-to-3'
oNL41	CTTTTGTCTTATACTTGGATCCTCTAGAGCCACCATGGAAGA
oNL42	CAACTAGAAGGCACAACTAGTTATGGCTGATTATGATCTAGAGTCG
oNL47	CTTTTGTCTTATACTTGGATCCCATGAGCGGCTACGTTAACA
oNL49	ATGGAGCACATACAGGGAGC
oNL50	CAACTAGAAGGCACAACTAGTTCACAGCTTCAGGAACGTCTTCC
oNL53	GATGTCGACCTTGTCATCGTCGTCCTTGTA
oNL56	GCTGGATCCACTGAATATAAACTTGT
oNL67	CAGGGTGTTGATGATGCCTTC
oNL74	GATCATGGCGCGCCTATGATGGCCAAGCCTTTGTC
oNL75	GTTCTTTGTACAGGCCCTCCCACACATAACC
oNL76	CATACGATGTTCCAGATTACGCT
oNL77	CATTCGATTAGTGAACGGATCT

#### BiLC expression constructs

A lentiviral vector (pRAR3G, TRE3G PURO) carrying all the sequences necessary for Tet-On 3G expression and puromycin selection was obtained as a generous gift from Inder Verma's lab. We digested the lentiviral backbone vector with BamH1 and SpeI restriction enzymes and purified the gapped vector. To insert K-Ras for expression as a BiLC fusion (pNL6-8), we performed PCR from CMV-promoted K-Ras BiLC fusion templates given to us by Geoffrey Wahl's lab: nLuc (1–416 aa of the *luc2* gene)-linker-HA-GGA-TCC-K-Ras either WT or G12D. The linker consisted of the following DNA sequence: 5'-GGAGGTGGATCTGGCGGAGGTCAGATCAGCTACGCCAGCCGGGGC-3'. PCR amplification was performed using oNL41 and oNL42 primers, and BamH1-Spe1-digested and purified products generated two fragments (BamH1-nLuc-HA-BamH1 and BamH1-linker-HA-GGA-TCC-K-Ras-Spe1), which were ligated into the BamH1-Spe1 lentiviral backbone vector in a 3-way ligation reaction, creating nLuc- linker-HA-GGA-TCC-K-Ras under the Tet-On 3G promoter for expression. To create nLuc (1–416 aa of the *luc2* gene)-linker-HA-GGA-TCC-K-Ras Y40C, BamH1-nLuc-HA-BamH1 was ligated to BamH1-linker-HA-GGA-TCC-K-Ras Y40C-Spe1, which was generated using digested PCR product, made using oNL42 and oNL56 primers for PCR amplification of the CMV-promoter cLuc-linker-HA-GGA-TCC-K-Ras-Y40C.

To subclone the cLuc (398–550 aa of *luc2* gene)-linker-Flag-GTC-GAC-Raf-RBD (1–149 aa) expression construct into the same lentiviral backbone (pNL9), oNL47 and oNL53 primers were used to PCR amplify cLuc-Flag-linker from CMV-promoter cLuc-K-Ras Y40C obtained from the Wahl lab to use as a template. This yielded a BamH1-cLuc-Flag-linker-Sal1 product, where the linker is 5'- GGAGGTGGATCTGGCGGAGGTCAGATCAGCTACGCCAGCCGGGGC-3'. The Raf-RBD (1–149 aa) region was PCR amplified using the GST-Raf-RBD plasmid obtained from Addgene (#13338) [57] as a template and oNL50 and oNL54 primers to generate Sal1-Raf-RBD-Spe1. The BamH1-Spe1 lentiviral vector was ligated with BamH1-cLuc-Flag-linker-Sal1 and Sal1-Raf-RBD Spe1 to create cLuc-Flag-linker-Sal1-Raf-RBD under the Tet-On 3G promoter for expression. The puromycin resistance cassette (PuroR) was removed by digestion with AscI and BsrG1 and replaced by amplifying the gene encoding Blasticidin S deaminase (BSD cassette) using oNL74 and oNL75, digesting the PCR product with AscI and BsrG1, and ligating this product to the cLuc-Flag-linker-Sal1-Raf-RBD plasmid digested with the same enzymes and lacking PuroR.

To verify the absence of unintended mutations within nLuc-HA-K-Ras plasmids (pNL6-8), each construct's ORF was sequenced in its entirety using oNL41, oNL42, and oNL67. The cLuc-Raf-RBD (pNL9) was sequenced in its entirety using oNL49 and oNL50 to ensure a lack of mutations.

#### Tag-BFP fusion protein

This lentiviral plasmid was a generous gift of the Wahl lab and is driven by the EF1α promoter with a start codon followed by a sequence encoding Gly followed by HA, a Gly Ser linker, followed by the TagBFP coding sequence, a Ser Gly linker, and the KRAS4B sequence encoding aa169-188 (UniProt P0116-2) for the hypervariable region and CAAX. The ORF is followed by a WPRE (Woodchuck Hepatitis Virus Posttranscriptional Regulatory Element). Sequences from the gifted plasmid were confirmed using oNL76 and oNL77 primers.

### Ultracentrifugation harvesting of EVs

#### Small EVs containing exosomes

This protocol was adapted from traditional exosome harvesting protocols [13,58]. Cells were grown in their usual medium for 24 hr. Medium was removed and replaced with fresh exosome-harvesting medium (containing exosome-depleted FBS for cells grown in FBS-supplemented medium). Exosome depletion of FBS was achieved by centrifuging FBS overnight at 110,000*xg* to pellet exosomes, and then FBS supernatants were sterile filtered with 0.2 μm filters. Cells were grown for 48 hr in exosome-harvesting medium, then conditioned medium was collected from plates and spun at 300*xg* to remove cells. Supernatants from the 300*xg* spin were spun at 16,500*xg* for 20 min to remove dead cells and cellular debris, and supernatants were then pooled and filtered using 0.2 μm filters to retain only smaller vesicles. Exosomes were harvested from filtered supernatants by spinning at 110,000*xg* for one hour and washing pooled samples in PBS. An additional 110,000*xg* spin for one hour in a smaller volume of PBS was performed to concentrate the exosomes for experimental analyses.

#### Small and large EVs

Cells were grown for 48 hr in exosome-harvesting medium, then conditioned medium was collected from plates and spun at 300*xg* to remove cells. EVs were harvested from supernatants by spinning at 110,000*xg* for one hr and washing pooled samples in PBS. An additional 110,000*xg* spin for one hour in a smaller volume of PBS was performed to concentrate the EVs for experimental analyses.

### Protein quantitation

Exosomes were permeabilized for 30 min on ice in 0.1% TX100 prior to quantitation. Cell lysates were diluted 1:5 for analysis. BSA standards were prepared using the appropriate buffer (0.1% TX100 or 20% lysis buffer). DC protein assay (Bio-Rad) reagents and protocol were used, and samples were calculated in parallel to a BSA standard series. All samples were plated in triplicate and averaged on 96-well plates and A750 readings were recorded on a TECAN M1000pro.

### Immunoblot analyses

Antibodies used in this study are listed in Table 3 along with their sources and part numbers.

**Table 3:** Antibodies used in this study.

Protein/tag detected	Source#
pan-Ras	Cell Signaling Technology #3965
tRFP (Tag-BFP)	Evrogen #AB233
Integrin α5	Cell Signaling Technology #4705
HA	Santa Cruz #SC-7392
Flag	Sigma #F3165
Tubulin	Sigma #T5168

Samples were resolved on 12.5% acrylamide-bis gels using standard protocols for SDS-PAGE, and gels were transferred overnight at 4°C with constant voltage (22 V) to PVDF membranes optimized for Odyssey detection. Li-Cor Odyssey protocols were followed for blotting of membranes with primary and secondary antibodies (Alexa dye 680 and IRDye 800), and after washing, these were scanned by the Li-Cor Odyssey for quantitative detection of signals. For quantitation, bands for proteins of interest were manually defined using a rectangle or ellipse, and an identical size/shape background band was manually defined (user defined background method). Normalized intensities were calculated from I.I. K counts given by the Odyssey software by subtracting background values from bands of interest and calculating this signal as a fraction of background-subtracted signal for a housekeeping protein.

For calculations of percentage release of Flag-K-Ras or nLuc-HA-K-Ras fusion proteins in exosomes, immunoblots were quantitated as described above. Cell-normalized equivalents were resolved by SDS-PAGE for whole cell lysates and for exosomes, but fewer cell equivalents were used for whole cell lysate than for exosomes (since the signal for fusion proteins in whole cell lysate is more concentrated). We tracked the number of cells per lane and the number of exosome producing cells that yielded the amount of exosomes loaded per lane. We multiplied the background subtracted, integrin α5-normalized values for fusion proteins’ signals (I.I. K counts) in whole cell lysates by a factor determined by dividing the number of cell equivalents in exosomes by the number of cell equivalents in whole cells. This scales up the intensity of the whole cell fusion protein to roughly normalize for cell equivalents between whole cell lysates and exosomes.

### Assay for vesicular uptake using DiD-labeled exosomes/small EVs

This protocol was adapted from a previous study [52]. Nf5310 cells were seeded at 10^5^ cells/ml in N2 medium supplemented with 1% exosome-depleted FBS onto chamber slides. Cells were grown for 48 hr. 005 cell exosomes (100 μg protein) or mock PBS samples were pelleted for one hr at 110,000*xg* and diluted into PBS Vybrant DiD dye (Thermo Fisher) added at ratio of 1:243. Samples were incubated for 15 min at 37°C, and then pelleted again for one hr at 110,000*xg*. Two washes in PBS were performed, pelleting each time at 110,000*xg*. Mock-labeled PBS or DiD-labeled 005 exosomes (at 50 μg protein/ml) were added to the media (N2 + 1% exosome-depleted FBS) of 2 wells of the chamber slide, and this was incubated for 30 min at 37°C, with gentle agitation every 10 min.

**Fig 1:**
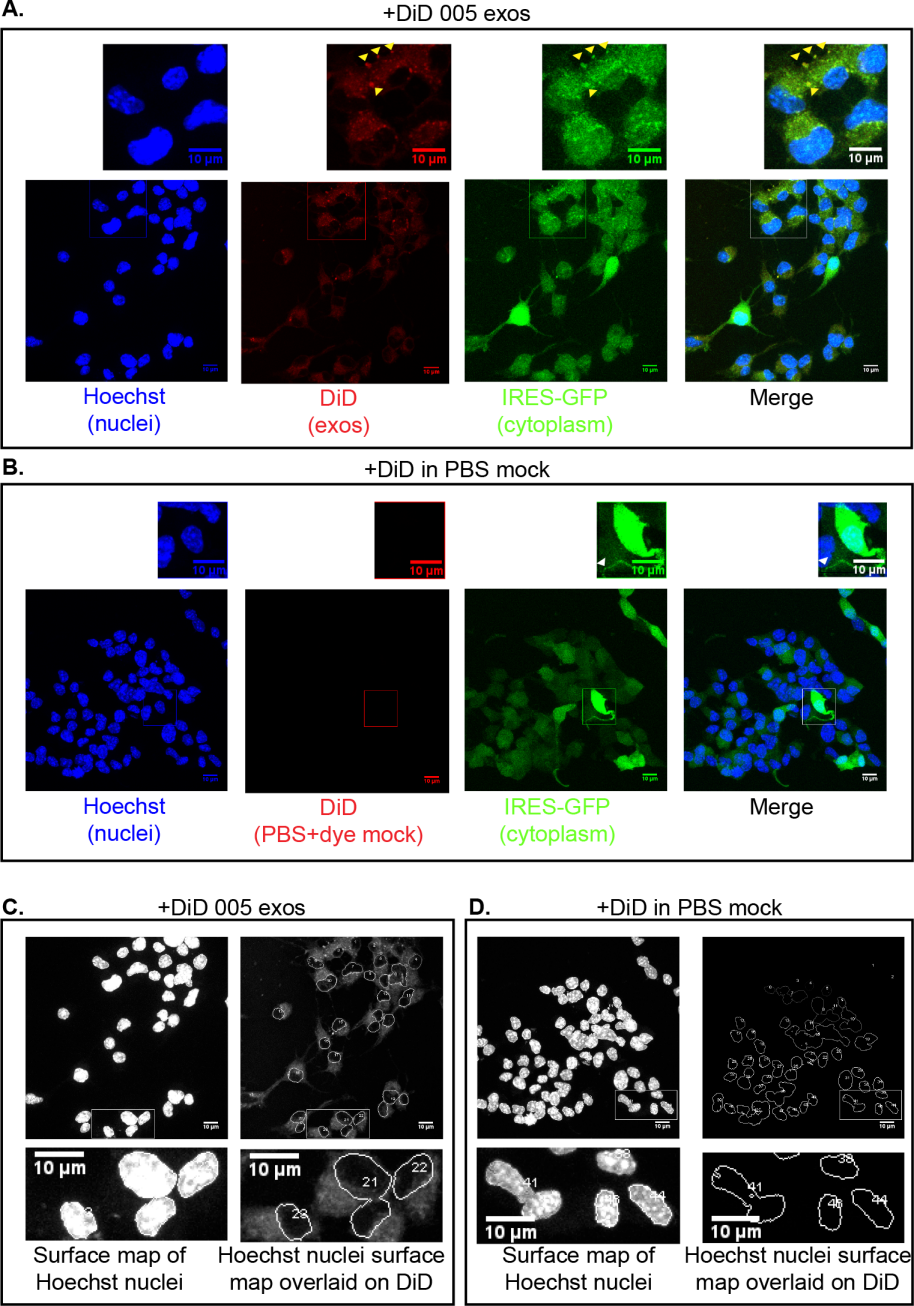
Dye-labeled exosomes transmit fluorescence to the cell cytoplasm. Confocal microscopy–single experiment-derived maximum projections of z-sections of fixed/permeabilized Nf5310 cells after 30 min uptake of DiD dye-labeled 005 GBM exosomes (exos) (A, C) or a DiD-treated PBS mock sample (B, D). (A, B) Each channel is represented separately or as a merge of all 3 channels. Images were cropped in the boxed regions with the resultant images displayed above to highlight aspects of localization. (A) Yellow arrowheads show puncta observed in both the GFP and DiD channels (co-localized), perhaps representing aggregates of 005 vesicles with DiD-labeled membranes and cytoplasmic GFP due to the IRES-GFP expressed in 005 cells. (B) The white arrowhead shows a small punctate structure that is visible on the GFP channel, but not the DiD channel fluorescence, emphasizing the lack of bleed through from the GFP channel. (C, D) Hoechst nuclei surface map overlaid on Hoechst channel (images on left) or overlaid on DiD channel fluorescence (images on right). Note that the punctate structure indicated by the white arrowhead shows only green signal, rather than yellow, due to the lack of signal in the far red channel, which detects DiD. Boxed areas for top images were cropped and resultant images are represented as images below to enhance visibility.

Following incubation, wells were washed 3x for 3 min in PBS, then fixed in 4% PFA for 10 min at room temperature. After fixation, samples were washed with PBS containing 0.1 M glycine for 10 min to block the remaining formaldehyde, and then rinsed twice more with this solution, followed by permeabilization for 5 min in 0.1% TX100 in 1X PBS. Following permeabilization, samples were washed 3 more times for 3 min in PBS. Hoechst 33342 stain was added for 5 min at 0.4 μg/ml, and slides were mounted to cover slips using Prolong Gold anti-fade (Thermo Fisher). Mounted slides were examined on an LSM 780 (Zeiss) microscope, capturing a z-series using a CMV-promoted IRES-GFP signal (488/543 Ex/Em) to designate the boundaries of the cytoplasm, using Hoechst 33342 signal (405/499 Ex/Em) to calculate the number of cells based on nuclei, and using the far red signal (DiD, 633/697 Ex/Em) to detect exosomes. Fiji (ImageJ 1.50a) [59] was used to generate the surface map of nuclei with the 3D object counter, eliminating edge objects [60]. Numbers of objects were loosely interpreted as number of cells (based on number of nuclei). The Hoechst surface map was overlaid (by merging channels in Fiji of the .tif files) with the DiD signal, and we determined whether all of the nuclei were touching signals for DiD (overlay of one representative image for control and experimental conditions shown in Fig 1C and 1D). Three images containing 117 cells were analyzed for the 005 exosomes (DiD) treated cells and for the mock treated cells (S1 Fig contains the DiD/nuclei surface map overlays not included in Fig 1).

### Cellular viability assays

Cells (1.5 x 10^5^ cells/ml) were plated in DMEM medium supplemented with exosome-depleted FBS (10%) and antibiotics on 96-well plates (100 μl) and incubated at 37°C in 5% CO_2_. After 24 hr, medium was replaced with 100 μl serum-free medium, with medium added to one well without cells for each sample as a control, and incubated for 5 hr. Ultracentrifugation purified exosomes in PBS (10 μl) were then added at the indicated concentrations to all wells, including controls, and cells were returned to the incubator. Following a 21 hr incubation, alamarBlue (Thermo Fisher) reagent was added (10 μl), and 560 nm (10 nm) excitation and 600 nm (20 nm) emission were recorded on a Tecan M1000pro for all time points indicated, with plates being incubated when not being scanned. Other settings used with the Tecan program were: Mode Bottom, Lag time 0 μs, Integration time 20 μs, Gain optimal, 400hZ, Optimal flashes per well, Settle time 0 μs, and default z-position. Three wells plus the control exosome/medium only well were used for each sample in each analysis. Values for the three wells were averaged in each experiment, with the exosome/medium only well value subtracted. Background-subtracted values were then averaged for all experiments (n = 7) and subjected to data analyses using R version 3.2.2 [61].

### Data analysis

For analyses of the effects of exogenous exosomes upon cellular viability, we used R version 3.2.2 [61]. By generating histograms of our data frames, we found that our data were not normally distributed, and so we selected a non-parametric test for unpaired data, the Mann Whitney test, to probe for statistical significance of our data comparisons. GraphPad Prism 7.0c corroborated the statistical significance of these data and was used for all graphs in this study.

### BiLC analysis

We followed established protocols for these experiments [62]:

#### Lysates

Used protein-protein interaction (PPI) lysis buffer (100 mM Tris-HCl pH 7.5, 0.5 mM EDTA, 150 mM NaCl, 0.1%Triton X-100, 1 mM sodium orthovanadate, 50 mM NaF), 100 μl per well on a 6-well plate. Cleared lysates were prepared from cells cultured for 40 hr on 6-well plates at the same density/conditions as for cultured cells BiLC analysis, and 20 μl lysate was diluted into 60 μl of DMEM without phenol red. Ten μl of the mixed lysates was pipetted into a 384-well plate (Corning, #3570) and incubated at room temperature for 10 min. Twenty μl of SteadyGlo (Promega) were added to each well, and luminescence read immediately with a Tecan M1000pro, integration time 0.5 sec, 3 min per cycle for 30 min at room temperature (no heating).

#### Cultured cells

A 384-well plate (Corning, #3570) was prepared with 2X concentrated reagents (400 μM D-luciferin, 0 or 500 ng/ml Tet) using DMEM (no phenol red) supplemented with 10% FBS and antibiotics, adding 20 μl per well. Cells were resuspended at 10^6^ cells/ml, counting viable cells using Trypan blue and a hemocytometer. Twenty μl per well were aliquoted in the 384-well plates already containing 2X concentrated reagents. Plates were sealed with MicroAmp Optical Adhesive Film (#4311971, Life Tech). Luminescence was read in a Tecan M1000pro set for 15 min cycles for 22 hr with an integration time of 2 sec at 37°C.

### Cellular uptake of EVs bearing Tag-BFP-HVR-CAAX

U87MG cells were cultured overnight in phenol-free DMEM: F12 +10% FBS media on 96-well plates at 7 x 10^5^ cells/ml. In the morning, medium was replaced with serum free phenol free medium (with no additional serum added throughout the remainder of the experiment), and cells were incubated for 5 hr prior to adding EVs. In parallel, a cell free medium culture was performed. After 5 hr of incubation, EVs (see ultracentrifugation protocols detailed above) were added at 20 μg total EV protein/ml in serum-free phenol-free medium at a volume of 10–20μl, and samples were incubated for the indicated time points. For analysis of remaining EV reporter protein in the supernatant, 15% of the supernatant volume was aliquoted into a tube containing the appropriate amount of 5X SDS-PAGE loading buffer (0.25 M Tris-Cl pH 6.8, 5% SDS, 0.05% bromophenol blue, 50% glycerol, 14% 2-mercaptoethanol). The remaining supernatant was removed, cells were washed once with 1X PBS-/-, and an equivalent volume of 1X SDS-PAGE loading buffer in phenol free medium was used to lyse the bottom of the well to yield cell lysates. Immunoblot analysis protocols were followed including 15% of the EVs input for indicated experiments.

For experiments using BafA1, this was added 1 hr prior to adding EVs (after 4 hr of serum starvation). The final concentration was 200 nM.

## Results

### Lipophilic dye-labeled GBM exosomes transmit fluorescence to GBM cells in culture

In a previous publication, we demonstrated that GBM exosomes are enriched for signaling proteins and contain active GTP-bound Ras, and that active Ras interacts with exosome proteins based on GST-Raf-RBD pulldown data [1]. In light of these results, we wondered whether GBM exosomes are taken up by other GBM cells to influence their signaling status. First, we investigated whether 005 mouse GBM exosomes could be taken up by other GBM cells by labeling exogenous exosomes with the fluorescent lipophilic dye, DiD (644/665 nm) which was used in a prior publication to assess uptake of Ras-bearing exosomes [52]. For this purpose, we used Nf5310 cells, a mouse GBM cell line established from tumors lentivirally targeted by shRNAs to NF1, p53, and PTEN (Verma lab, unpublished results). These cells adhere well to chamber slides for immunofluorescence analysis (more so than 005 cells) and also express GFP cytoplasmically by virtue of an IRES-GFP carried by the tumor-initiating lentiviral vector.

To test the ability of these cells to take up 005 exosomes, we compared the ability of Nf5310 cells to take up 005 exosomes labeled with DiD dye or PBS treated with DiD dye as a control, using a 30-min incubation at 37°C in 5%CO_2._ We utilized laser confocal microscopy (Zeiss LSM 780) to capture z-series of the cytoplasm (visualized by IRES-GFP) and the nucleus (visualized by Hoechst 33342 dye) to examine whether exosomes could be taken up into the cytoplasm or nuclei of cells.

In a single experiment (Fig 1), we confirmed results reported in a prior publication examining Ras-bearing exosomes [52]. Using a time point at which 100% internalization of exosomes was observed in this study (30 min), we captured z-series from fixed/permeabilized cells. Maximum projections of these images revealed DiD signal for exosomes labeled with DiD, but not for PBS treated with an identical concentration of DiD dye as a mock control (compare DiD signal in Fig 1A to Fig 1B). Cropped images for cells treated with DiD-labeled exosomes (top image, Fig 1A) display puncta in the far right channel (DiD) that co-localize with GFP (yellow arrowheads), perhaps representing aggregates of 005 vesicles with DiD-labeled membranes and GFP-labeled vesicular cytoplasm (since 005 cells also express cytoplasmic GFP through an IRES). To confirm that free dye does not cause channel bleed-through, we cropped a region that contained strong GFP fluorescence for mock- (PBS+DiD dye) treated cells (bottom image, Fig 1B). No fluorescence was observed in the channel for the DiD signal, and a white arrowhead depicting a small punctate structure present on a cropped image for the GFP channel was not discernible in the cropped region in the far red channel depicting DiD (top image, Fig 1B). Furthermore, the punctate structure was green rather than yellow on the cropped, merged image (white arrowhead, top image, “Merge”), emphasizing that DiD signal is not merely a reflection of GFP signal bleed-through.

To quantitate the number of cells and the presence of signal in an unbiased manner from maximum projection data of images, we used the 3D object counter [59] on Fiji ImageJ [59] to generate a surface map of nuclei based on Hoechst signal overlaid onto the far red channel signal for DiD-treated exosomes (Fig 1C) and onto DiD-treated PBS (Fig 1D). We used nuclei to determine the number of cells rather than GFP, since GFP would bias the data towards Nf5310 cells with greater expression and/or integration of the IRES-GFP carried on the original glioblastoma tumor-initiating lentiviral vector. The 3D object counter provides a number for each nucleus identified. Each nucleus was scored for the presence of adjacent DiD signal (from the associated cytoplasm) for a total of 117 cells in both the control and experimental groups compiled from 3 images for each. Fig 1C and 1D show detail for 1 of the 3 images; S1A and S1B Fig provide the remaining 2 images represented as surface maps of nuclei merged with the far red channel. All of the cells treated with 005 GBM exosomes labeled with DiD yielded visibly detectable signal for the cytoplasmic area surrounding each nucleus (Fig 1C, S1A Fig), but no detectable signal was observed surrounding nuclei of cells with mock-treated PBS (Fig 1D, S1B Fig). Cropped images also highlight the predominant cytoplasmic localization of the DiD signal (Fig 1C, compare surface map on nuclei to surface map of the DiD signal).

These results demonstrate that fixed, permeabilized U87MG cells display fluorescent signal in the cytoplasm after prior incubation for 30 min with dye-labeled U87MG exosomes. The presence of what appeared to be aggregates of 005 vesicles (displaying DiD signal and GFP signal) within the Nf5310 cells’ cytoplasm supported the idea that 005 exosomes were transferred into the cytoplasm of Nf5310 cells. The lack of signal in the DiD channel, even in areas of high GFP expression emphasizes the lack of bleed through from the GFP channel to the far red channel. Since lipids (not proteins) are labeled in this experiment, and the cytoplasm of both Nf5310 and 005 cells expresses GFP, it is unclear from these results whether exosomes empty their contents into the cytoplasm of acceptor cells for functional reuse. Thus, additional experiments are necessary to determine whether exosomes have indeed transferred contents into the cell’s cytoplasm and whether exosome contents can be functionally recycled within the accepting cells.

### Treating serum-starved GBM cells in culture with exogenous GBM exosomes improves cell viability

Since exosomes contain signaling proteins and active Ras, which regulate cellular proliferation and viability, we wondered whether exosomes containing these factors can be taken up by cells to functionally impact these processes. One method that has been used to synchronize signaling pathways of cells is to remove growth factors from the medium by serum-starving cells followed by growth factor stimulation (e.g. EGF) to study effects on a specific signaling pathway, such as Ras/Raf/MEK/ERK. We employed serum starvation to examine whether exogenous exosomes added to serum-starved cells would improve their proliferation/survival over time, expecting that these could perhaps stimulate Ras and other signaling pathways based on exosome proteome enrichment for signaling proteins. For these experiments, we used U87MG human GBM cells and exosomes, since we expected to characterize any functional effects in future experiments through stable transduction of dominant negative and constitutive mutant forms of K-Ras (005 GBM cells are already stably transduced with Flag-H-RasV12, precluding their use for these studies).

**Fig 2:**
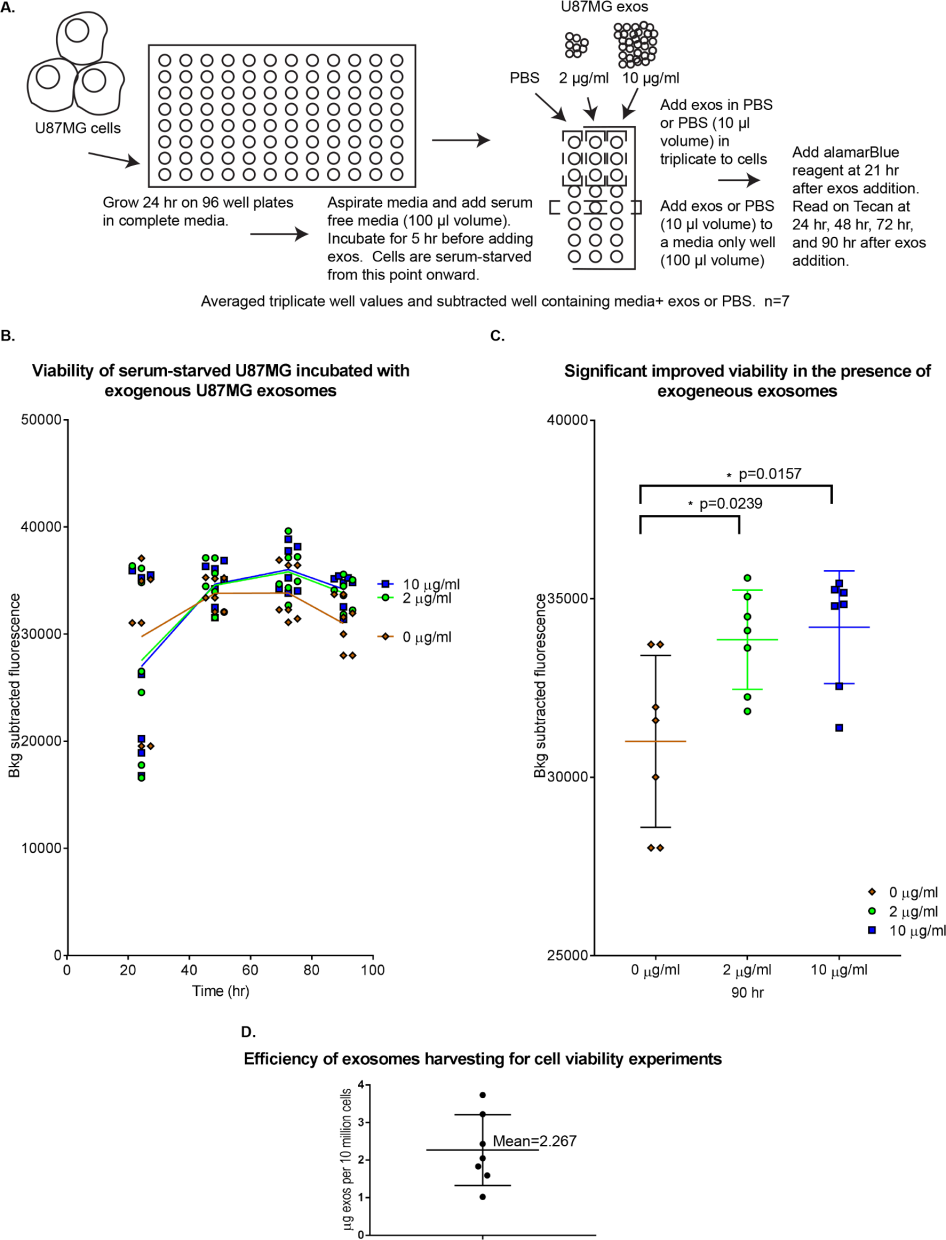
Exogenous exosome treatment improves viability of serum-starved U87MG cells. (A) Cartoon/description of the experiment. (B) XY scatter plot of background-subtracted fluorescence values (an average of triplicate technical replicates) plotted against time for each independent experiment (n = 7), with means connected by a line; spreadsheet containing background-subtracted values for 24, 48, 72, and 90 hr time points is available as S1 Table. (C) Detail of 90 hr time point: Scatter dot plot with means and error bars for standard deviation; Mann Whitney two-tailed test performed at p>0.05 level of significance on GraphPad Prism to yield * = significant result. Exact p-values are given for each comparison. (D) Column graph to show efficiency of exosome harvesting (μg of exosomes obtained from 10^7^ cells) for exogenous exosomes used in cell viability experiments in (B) and (C) (n = 7). Individual replicate values, mean and error bars for standard deviation are shown. Exos = exosomes.

To probe for effects of exosomes on proliferation of serum-starved cells, we cultured U87MG cells on 96-well plates. After serum starving established cells for 5 hr, U87MG exosomes were added at the indicated concentrations, and cell viability was measured using alamarBlue over a time course of 90 hr (see Fig 2A for a cartoon/description), with serum starvation continuing throughout the entire time course of the experiment.

The graph demonstrates that 24 hr after adding exosomes, cells growing without serum but with either 2 μg/ml or 10 μg/ml exosomes grew similarly on average to the control cells, recording means that are slightly higher in the control cells but without statistical significance (Fig 2B, S2 Fig, S1 Table). The variation in alamarBlue background-subtracted values is similar for each group (S2 Fig, S1 Table). Since equal amounts of cells were seeded at the beginning of the experiment, and 3 wells were used as technical replicates to average for each time point in this experiment, we suspect that there might be variability in cell number as the cells adjust to serum starvation, albeit the variability is similar across the tested groups, However, at 48 hr and at 72 hr, there was less variability in the recorded values of the samples, and no statistically significant relationships were observed. By 90 hr, cells with exogenous exosomes added at 2 μg/ml or 10 μg/ml demonstrated a significantly improved viability over the 0 μg/ml control (p-values of 0.0157 and 0.0239, respectively, using the Mann Whitney test at a p>0.05 level of significance on GraphPad Prism version 7, Fig 2C), increasing viability on average by 9% and 10%, respectively (S1 Table, “Mean values”). The downward trend of the means after 72 hr indicates that the improved cell viability observed at 90 hr for exogenous exosome-treated samples is unlikely to be attributed to increased proliferation but might represent increased survival under nutrient limitation.

Overall, these results suggest a functional impact of exosomes upon cells during serum starvation, although additional data are required to determine whether this phenotype is a consequence of exosome internalization, as opposed to the interaction of exosome-derived components with receptors on the cell surface (see Discussion).

It is also important to consider the concentrations of exogenous exosomes used in these experiments. The efficiency of exosome harvesting from cells for these experiments is plotted in Fig 2D. At the lowest concentration used (2 μg/ml), exosomes harvested over 48 hr of growth from 880,000 cells, on average, were incubated with 15,000 recipient cells. These conditions are unlikely to reflect the total amount of exosomes released under physiological conditions (see Discussion), and so the phenotypes observed with added exosomes might not reflect a physiologically relevant mechanism. Therefore, to understand whether Ras is directly exerting an effect upon cell viability through intercellular transfer, we developed a system to probe the ability of K-Ras in exosomes to interact with a downstream signaling protein (Raf-RBD) in acceptor cells.

### Bimolecular luciferase complementation (BiLC) fusions can be expressed to probe for intercellular transfer in cultured cells

We considered that intercellular transfer of active Ras via exosomes (or other mechanisms) might occur to promote stress resistance within a population of cells. If active Ras is directly transferred from one cell to another, donated Ras should be capable of interacting with the Raf kinase via the Raf-RBD in the acceptor cell.

**Fig 3:**
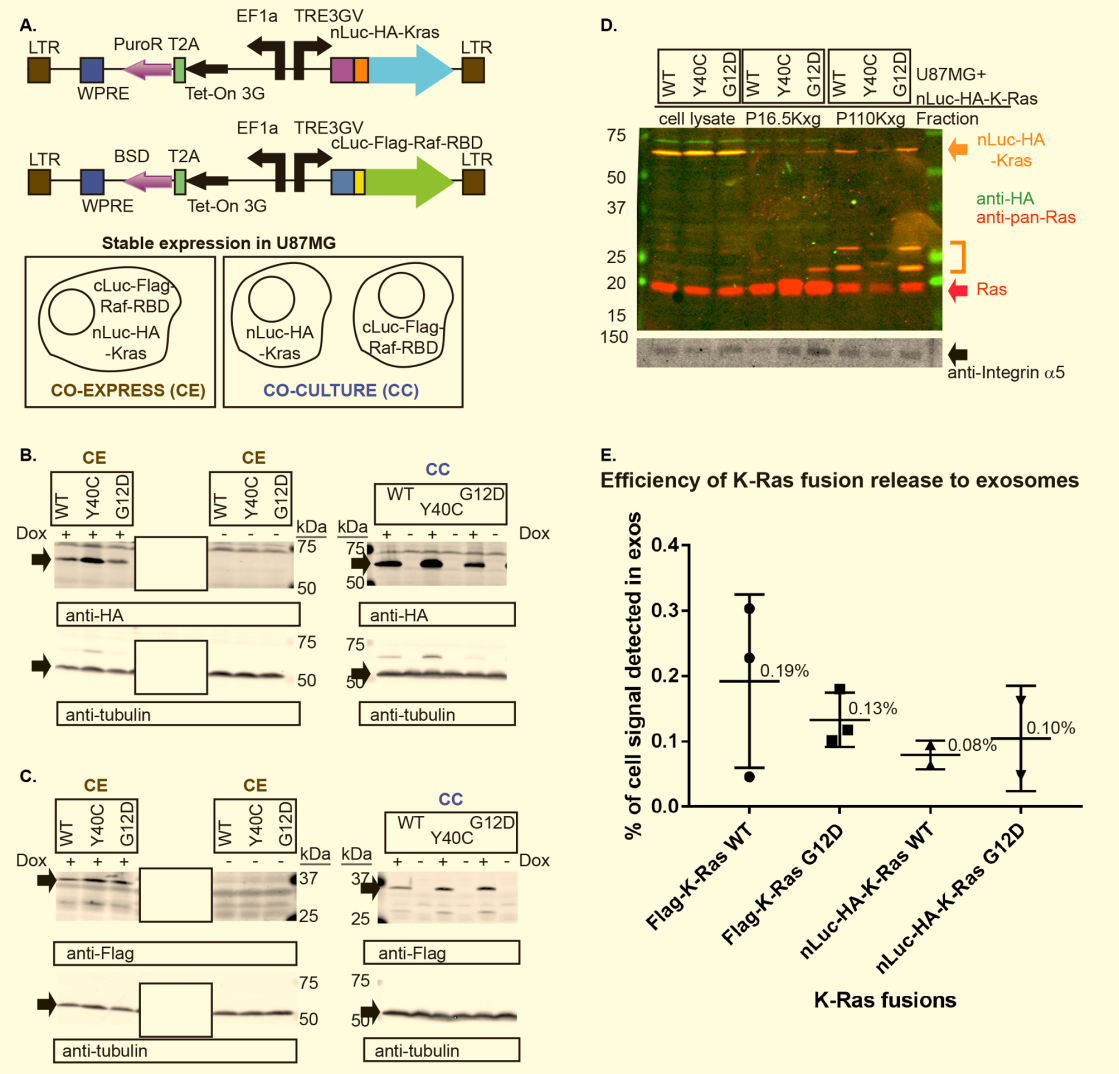
BiLC fusions are expressed. (A) Map detailing lentiviral expression constructs for BiLC fusions, stably expressed in U87MG cells. Cartoon depicts how these fusions were stably expressed in cells for co-expression (CE) and co-culture (CC) experiments. LTR = long terminal repeat, WPRE = woodchuck hepatitis virus post-transcriptional regulatory element, T2A = self-cleaving peptide, PuroR = puromycin resistance cassette, BSD = blasticidin resistance cassette, Tet-On 3G = transactivator protein, EF1a = translation elongation factor 1a promoter, TRE3GV = Tet-On 3G system promoter. (B, C) Lysates from cell-normalized equivalents from co-cultured (CC) or co-expressed (CE) BiLC fusions stably expressed in U87MG and grown in media +/- Dox (250 ng/ml) (using conditions detailed for the BiLC experiment in Materials and Methods) were resolved on an SDS gel and immunoblotted for (B) nLuc-HA-K-Ras (anti-HA) or (C) cLuc-Flag-Raf-RBD (anti-Flag) with tubulin levels used for normalization (black arrows indicate protein of interest). K-Ras mutations analyzed are indicated above lane(s). Irrelevant lanes on the same gel are covered with a box. Complete, uncropped blots are available in S3 Fig (D) BiLC K-Ras fusion protein expression in lysates from 1.0 x 10^5^ cells (cell lysate), and from lysates of pellets of conditioned media: produced from 3.7 x 10^7^ cells (16,500*xg* pellet = P16.5Kxg) and from 2.1 x 10^7^ cells (110,000*xg* pellet after removing 16,500*xg* pellet and 0.2 μm filtration = P110Kxg). Color-coded arrows show protein bands of interest from a scan of an immunoblot probed with the indicated antibodies on the Li-Cor Odyssey using secondary detection antibodies labeled with two different colors. Integrin α5 was used as a normalization control. Bracket shows truncated fragments of full-length fusion protein. Separate channel images in grayscale are available in S3 Fig (E) Total background-subtracted signals for the indicated K-Ras fusion protein’s expression in exosomes purified from U87MG cells stably expressing the indicated constructs were normalized to integrin α5 signal. Whole cell lysate signals were calculated in the same manner, then multiplied by a factor to adjust for the diluted number of cells represented by this signal as compared to exosomes. Normalized exosome signals/whole cell lysate signals x 100 produced the percentages for each data point. Individual experimental data points are indicated as symbols. For nLuc-HA-K-Ras analysis: (D) and an additional independent experiment were used (a total of 2 independent experiments); calculations available in S2 Table. For Flag-K-Ras analysis: 3 independent experiments were used from a prior publication characterizing Ras release to exosomes [1]; calculations available in S3 Table.

We directly probed for functional intercellular transfer of K-Ras in live, cultured cells, by leveraging a recently published method describing a system for testing protein-protein interactions in cells growing on 384-well plates using tetracycline-inducible expression of proteins fused to complementing regions of the *luc2* gene encoding firefly luciferase, and subsequently probing for Bimolecular Luciferase Complementation (BiLC) in the presence of D-luciferin substrate [62]. Intracellular BiLC has been reported for bidirectional tetracycline-inducible expression of fusions to K-Ras and the Raf-RBD, using K-Ras Y40C as a negative control (personal communication, Wahl lab). We subcloned WT, Y40C, and G12D versions of nLuc (1–416 aa of firefly luciferase)-HA-K-Ras and the cLuc (398–550 aa of firefly luciferase)-Flag-Raf-RBD fusions derived from the Wahl group's constructs into the vectors described in Fig 3A, to create individual expression constructs with selectable markers under a unidirectional Tet-On 3G system promoter. Stable U87MG cell lines were created that expressed nLuc-K-Ras forms and cLuc-Flag-Raf-RBD separately for co-culture (CC) experiments using single drug selection or co-expressed (CE) within a single cell line that is puromycin and blasticidin resistant (see cartoon in Fig 3A). Dox-dependent induction of all BiLC fusions was confirmed under co-expressed and co-culture conditions in cells (Fig 3B and 3C), and the high speed pellet representing small extracellular vesicles (EVs) or exosomes clearly contained full-length BiLC K-Ras fusions (Fig 3D, orange arrow), albeit truncated forms of the fusion were evident in these preparations (orange bracket).

Since we had observed what appeared to be N-terminally truncated fragments lacking nLuc (Fig 3D, orange bracket), we examined the efficiency of BiLC K-Ras fusion release (for both wild-type and G12D) in exosomes as a percentage of total cellular levels of fusion protein expression. For comparison, we included data from our previous study of K-Ras release in exosomes in which the minimally-tagged Flag-K-Ras was used [1]. We quantitated the percentage of cellularly expressed fusion proteins released in exosomes, by first normalizing against integrin α5 levels, and then factoring in the number of cells from which exosomes were produced. Calculations are available in S1 and S2 Tables for nLuc-HA and Flag K-Ras fusions, respectively. These results demonstrated that fusion proteins were released in exosomes on average at a range of 0.08–0.19%. Although the longer (nLuc-HA) tag did decrease the average percentage of fusion protein released from cells in exosomes, the percentage release for individual experiments was in the range of the values observed for the release of 1X Flag-tagged K-Ras proteins in exosomes (Fig 3E). Hence, even though truncation fragments were observed in exosomes from cells expressing BiLC fusions, for both wild-type and G12D K-Ras fusions, the efficiency of release in exosomes was similar, albeit less on average, to Flag-K-Ras fusions.

### Functional complementation between K-Ras and Raf-RBD fusions expressed in separate cell lines is not detectable

**Fig 4:**
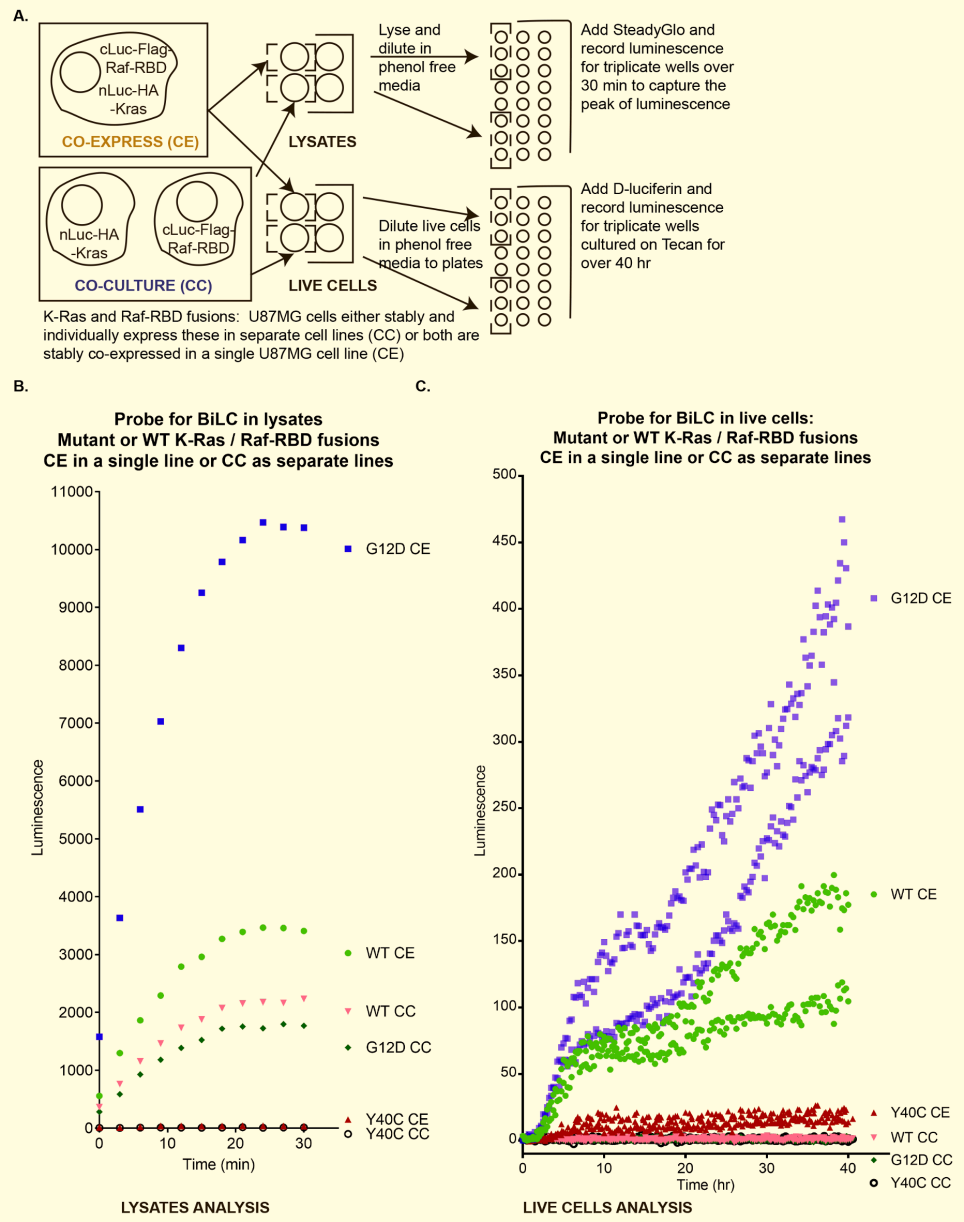
K-Ras and Raf-RBD fusions complement only in lysates or when co-expressed in cultured cells. (A) Cartoon / description of BiLC experiments. (B) Scatter plot for a single analysis showing averaged triplicate values for luminescence increasing over time for BiLC lysates from cells co-expressing Raf-RBD fusion with the indicated K-Ras fusion or co-culturing fusions as separate cell lines. (C) Scatter plot of 2 independent experiments (each with averaged triplicate values) using live cells to probe BiLC as in (B) but using cells cultured on plates over time.

To validate the functionality of these expressed protein fusions prior to probing for intercellular transfer of proteins, we examined their ability to exhibit BiLC in lysates where transfer across membranes is unnecessary. Accordingly, we prepared lysates from U87MG cells stably expressing K-Ras or Raf-RBD BiLC fusion constructs growing them separately (CC, 1:1 ratio of the U87MG cell line stably expressing nLuc-HA-K-Ras to the U87MG cell line stably expressing cLuc-Flag-Raf-RBD) or co-expressed (CE) within a single cell line (see cartoon in Fig 4A, “LYSATES”). Over the course of 30 min, a period sufficient to detect the peak of luminescence for cells co-expressing cLuc-Flag-Raf-RBD with nLuc-HA-K-Ras wild-type or G12D controls, we found that lysates from co-cultured cells exhibited luminescence in a manner dependent on an intact effector domain, since no luminescence was observed for cells co-cultured with the nLuc-HA-K-Ras Y40C effector domain mutant (Fig 4B). Cells expressing both Ras and Raf-RBD fusions in a single cell line yielded stronger, but also effector domain-dependent, luminescence (Fig 4B), with co-expression of K-Ras G12D with Raf-RBD fusions giving the greatest level of signal. Although wild-type K-Ras fusion yielded a greater signal when co-expressed with the Raf-RBD fusion in a single cell line rather than in co-culture as separate cell lines, the difference between co-culture and co-expression was less pronounced (Fig 4B) than for the G12D fusion.

We then asked whether intercellular transfer of K-Ras occurs in cultured cells, culturing U87MG cells stably expressing the BiLC fusions as a co-culture of separate cell lines and comparing this to co-expression of both K-Ras and Raf-RBD fusions within a single cell line (see cartoon in Fig 4A,”LIVE CELLS”). Over a 40 hr time course, cells co-expressing cLuc-Flag-Raf-RBD with nLuc-HA-K-Ras wild-type or G12D demonstrated increasing BiLC (Fig 4C). By contrast, results of this experiment failed to show BiLC for any of the co-cultured cell line analyses in as much as luminescence was undetectable and was even below that of the single cell line co-expressing nLuc-HA-K-Ras Y40C with cLuc-Raf-RBD (Fig 4C).

### Fluorescent reporter protein fused to the membrane-anchoring region of K-Ras remains predominantly in cell culture supernatants in cellular uptake assays

**Fig 5:**
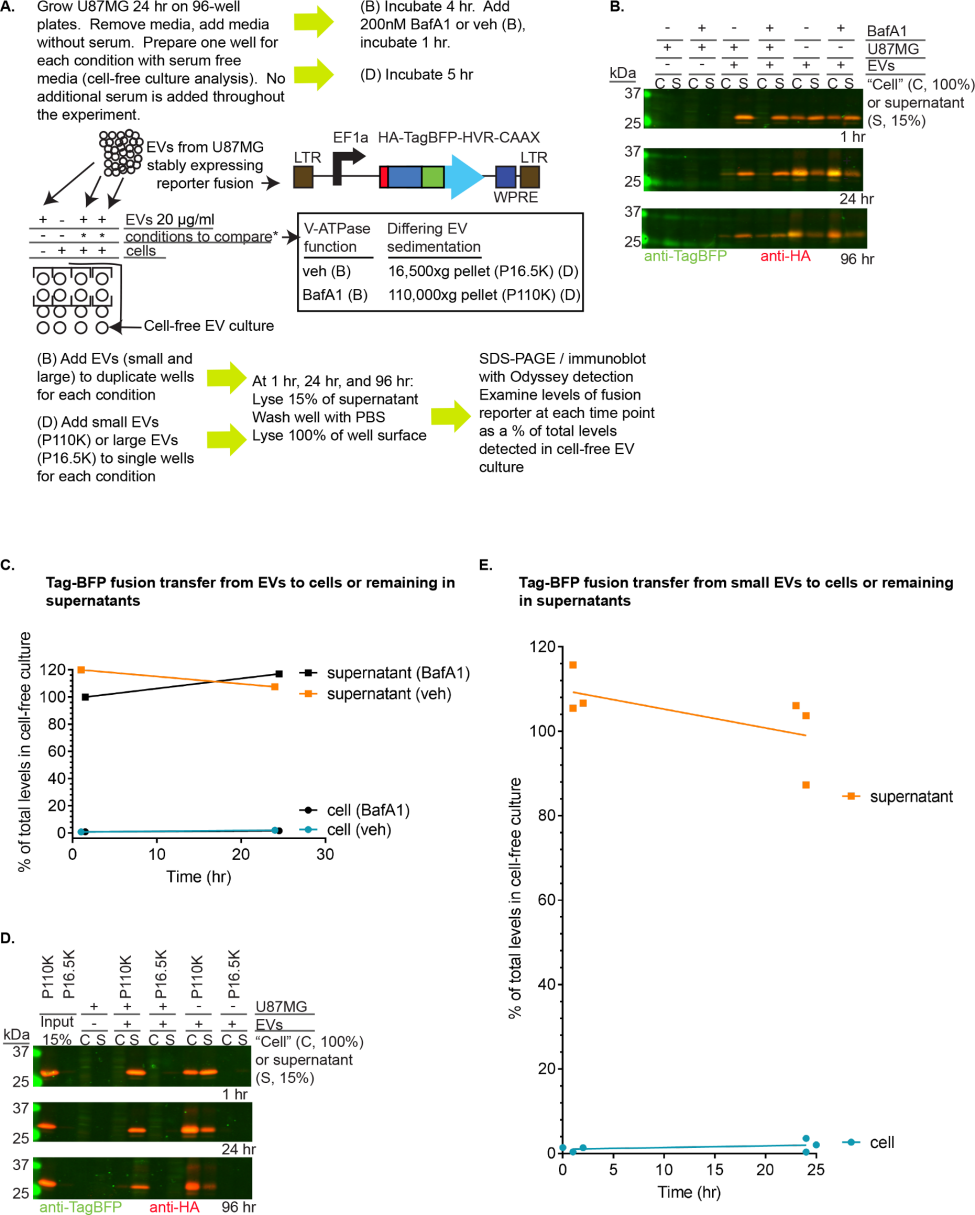
Fluorescent reporter protein remains primarily in the supernatant in assays for EV-to-cell transfer. (A) Cartoon / description of experiments to probe uptake of EVs by U87MG cells, examining small EV uptake +/- bafilomycin A1 (BafA1) (B) or comparing small EV uptake to larger EV uptake (D). LTR = long terminal repeat, WPRE = woodchuck hepatitis virus post-transcriptional regulatory element, EF1a = translation elongation factor 1a promoter (B), U87MG cells were seeded and grown 24 hr on 96-well plates at which point the media was replaced with serum free media (no additional serum was added for the remainder of the experiment), and wells of serum-free media were aliquoted to use as a cell-free culture control. The plate was incubated for 4 hr, and cells and cell-free media wells were treated with 200 nM bafilomycin A1 (BafA1) or a vehicle control (+/-). The plate was returned to the incubator for 1 hr, then EVs (small and large EVs, see “Materials and Methods”) were added to a concentration of 20 μg/ml. Two wells were used for each condition (technical replicates). At 1 hr, 24 hr, and 96 hr, samples were collected from the conditioned media (cell supernatant, S), and 15% of the total supernatant was resuspended with 5X SDS-PAGE loading dye to lyse. The bottom of the wells containing adhered cells (“cells” = C) were washed 2X with PBS, and cells were removed by suspending in a volume of 1X SDS-PAGE loading buffer equivalent to samples prepared for the supernatant. Lysates of 15% of the supernatant (S) and 100% of the well bottom (“cells” = C), for the two technical replicates, were resolved by SDS-PAGE and were immunoblotted as indicated. (C) HA-TagBFP-HVR-CAAX signals were quantitated from the immunoblot (B) and from the replicate not shown in (B), subtracting background from recorded intensities and normalizing the “cell” signals (multiplying by 0.15) for comparison to background subtracted supernatant signals (which were loaded at 15%). Cell co-culture signals for supernatant or “cell” were then divided by the total signal detected in cell-free co-culture with EVs (background subtracted supernatant signal summed with 15% of background-subtracted “cell” signal) to give a percentage. This percentage represents the amount of the fusion protein remaining in the media (supernatant) or associated with cells (either on the plate, on the cell surface, or inside of cells) when co-cultured with cells as compared to the total amounts of fusion protein detectable at the time point analyzed for cell-free culture. Scatter plot of signals in (B) showing percentage of total Tag-BFP fusion (total detected in cell-free co-culture) for cell and supernatant fractions at 1 hr and 24 hr time points for this experiment. (D) SDS-PAGE of lysates of supernatant or “cell” fractions from cells or media cultured with EVs for time points as in (B)but large EVs (P16,500*xg*) or small EVs (P110,000*xg*) fractions were separately incubated with cells or media, and BafA1 treatment was not analyzed. (E) Calculations as in (C) for P110,000*xg* Tag-BFP fusion uptake at 1 hr and 24 hr (n = 3 independent experiments). The lines show the position of the means.

Since we had observed lipophilic dye transfer in exosome uptake assays, yet we failed to observe complementation of the K-Ras / Raf-RBD interaction from cell-to-cell, we wondered what percentage of an EV protein remains in the cell culture supernatant as compared to the amount transferred to cells. To answer this question, we stably expressed a TagBFP reporter bearing the hypervariable region (HVR) and CAAX sequence of K-Ras in U87MG cells (experiments detailed in Fig 5A). We treated serum-starved naïve U87MG cells with exogenous EVs from the reporter-bearing strain, isolated using a protocol that should capture both small and large EV populations for a more global analysis. In addition, we performed the same experiment on cells treated 1 hr prior to EV addition and throughout the entirety of the experiment with the vacuolar H+-ATPase inhibitor (v-ATPase) bafilomycin A1 (BafA1) at 200 nM as compared to a DMSO vehicle control (veh). This condition was utilized to test whether altering v-ATPase function increases recovery of EV proteins taken up by cells.

Lysates from cells (harvested from the bottom of the plate after a PBS wash using 1X SDS-gel loading buffer) and cell supernatants (15% of the medium removed from each well prior to aspiration and PBS wash to add to concentrated loading buffer for a final concentration of 1X) were collected at 1, 24, and 96 hr, and 100% of the cell lysates was loaded and run next to cell supernatant lysates loaded at 15%, in order to enhance the ability to observe any signal for reporter proteins taken up by cells. To compare total levels of the reporter at each time point, eliminating the contribution of cells, EVs were cultured in parallel under cell-free conditions with lysates collected from supernatants and from the plate surface (“cells”) and loaded similarly to other samples. The amounts of reporter protein transferred to cells and remaining in the supernatant were calculated and expressed as a percentage of total reporter protein (calculated from cell-free culture of EVs at each time point) for 1 and 24 hr. The 96 hr time point was included for qualitative analysis only since constant volumes of media could not be maintained for 96 hr. Results of this experiment are demonstrated in Fig 5B.

Although in cell-free culture, the EVs appeared to adsorb to the wells based upon an increase in plate surface localization of the fusion protein over time (Fig 5B, see Discussion), the total levels of reporter protein in cell-free culture (“cell” signal added to supernatant signal) were used to calculate the percentage distribution of the fusion protein for samples including cells and EVs. The results reveal that the majority of reporter protein was found in the supernatant from 1 to 24 hr, with or without BafA1 treatment. After 1 hr of incubation, yields of the reporter protein in the supernatant were nearly 120%, greater than the total levels detected in cell free culture at this time point (Fig 5C), yet less than 1% of the reporter protein was detected in the cell lysates (Fig 5C). After 24 hr, levels of reporter protein increased to 1.9% in the cells, with a decrease in the supernatant levels to 107.4% (Fig 5C). Interestingly, inhibition of v-ATPase activity using BafA1 exerted little impact on the percentage of reporter protein in cells since at 1 hr (0.7% vs. 0.8%, Fig 5C), and at 24 hr, less reporter protein was detected in BafA1-treated cells as compared to controls (1.9% vs. 1.5%, Fig 5C).

In qualitative analysis, the EV reporter protein remained enriched in the supernatant rather than increasing in the level associated with cells at 96 hr. Although we did not quantitate the levels of reporter protein for samples incubated for 96 hr due to evaporative loss of supernatant volumes, visual analysis did not reveal a marked increase in reporter protein recovered from the cell lysates (Fig 5B). Moreover, signals for the EV reporter protein were more prominent in the supernatant lysates as compared to cell lysates, even without accounting for differences in percentage representation (100% for cell lysates and 15–100% for supernatant lysates due to media evaporation).

We expected that small EVs (which include exosomes) might impart a more enhanced uptake from vesicle-to-cell. To answer this question, we pelleted larger EVs at 16,500*xg* and then isolated smaller EVs after 0.2 μm filtration by pelleting at 110,000*xg*. These fractions were co-cultured separately as in the previous experiment using a single preparation of small and large EVs, and 15% of the input was included on the gel to readily compare to 15% of the supernatant lysate loaded on the same gel. Again, we loaded 100% of the cell lysate taken from the surface of the plate to enhance our detection of cells taking up the EVs.

The EV reporter protein was only detectably expressed in the P110,000*xg* pellet (Fig 5D). Results of this experiment for small EVs including exosomes (the P110,000*xg* pellet) were similar to that of the experiment using a single preparation of both small and large EVs (Fig 5B). Thus, we performed two additional independent experiments using small EVs only (the P110,000xg pellet) to quantitate levels of the EV reporter protein transferred to cells as compared to remaining in the supernatant, continuing to monitor these levels as a percentage of totals calculated for cell free culture at each time point, quantitating 1 hr and 24 hr time points.

The results of these 3 experiments analyzing reporter fusion transfer from small EVs to cells demonstrate that on average, 0.877% of the total EV reporter protein was found in the cell lysates after 1 hr, and this average more than doubled to 1.993% at 24 hr (Fig 5E). Nevertheless, nearly 100% of the amounts of EV reporter protein observed in cell-free culture were retained in the supernatant after 1 hr of EV co-culture with cells in each experiment (106.1% on average), and the supernatant continued to retain greater than 80% of EV reporter protein at 24 hr as compared to cell-free culture at the same time in all experiments (99.03% on average) (Fig 5E). Moreover, even at 96 hr, the signal for the EV reporter protein was qualitatively greater in the supernatant as compared to the cell lysate (Fig 5D). Thus, any transfer of the EV reporter protein from vesicles to cells is minor in comparison to the amount of EV reporter protein remaining in the supernatant.

## Discussion

Here, we present the results of a direct test of the ability of K-Ras protein to be transferred in extracellular vesicles from one cell to another to interact with the Raf RBD in a target cell and engage Ras pathway signaling in an intercellular manner. Although other publications have reported the effects of exogenous treatment of cells with Ras-bearing extracellular vesicles [52,63], direct evidence for Ras protein transfer into other cells to exert a function is lacking. We adapted a BiLC protein-protein interaction reporter system, which detects the K-Ras/Raf RBD interaction by luminescence in living cells and enables continuous analysis of co-cultured cells on multi-well plates. Using co-cultured cells stably and separately expressing K-Ras and Raf-RBD BiLC fusions, this system monitors K-Ras release and uptake from cell-to-cell (through any mechanism) in real-time, once the detection threshold has been achieved.

We initially hypothesized that exosomes could be exchanged between cells within a heterogeneous population of tumor cells, perhaps transferring signals intercellularly that support survival in the face of stress. Consistent with the work of others [52,64], we detected transfer of lipophilic dye-labeled small EVs into GBM cells (Fig 1). Moreover, we observed a significant improvement in cell viability during serum starvation upon treatment with exogenous exosomes (Fig 2). However, we were unable to detect direct, functional, intercellular transfer of K-Ras protein from one cell to another using the BiLC complementation assay, even though complementation was observed in lysates made from co-cultured cells separately expressing K-Ras and Raf-RBD fusions or in cultured cells co-expressing the fusions (Fig 4).

Indeed, studies of a fluorescent EV reporter protein fused to the HVR and CAAX motifs of K-Ras demonstrated that comparing culture of EVs containing this fluorescent protein in the absence of cells to culture with naïve U87MG cells, no more than 2% was transferred to cells attached to the plate (Fig 5). The amount of EV reporter protein transferred to the inside of cells is likely lower than this 2% value, since some EV reporter protein detected in cell lysates is likely due to EVs attached to plates or to the surface of cells. Notably, cell-free culture of EVs revealed that the surface of the plate contains associated EV reporter protein, either due to growth of cells not eliminated in the EV preparation protocol (possible in whole EVs experiments without filtration [65], but unlikely in 0.2 μm filtered small EVs experiments) or due to adsorption to the plate surface (Fig 5). Overall, it seems likely that if EV protein is transferred into cells this is likely to occur at a level less than 2% of input, which could lie below the threshold of detection in the BiLC assay.

Nevertheless, we did observe transfer of signal from lipophilic dye (DiD)-labeled vesicles into unlabeled cells (Fig 1) as early as 30 min, although the percentage of transfer was not calculated in these experiments. A mock control using the same amount of dye in PBS did not transfer signal to cells. This experiment may have a greater capacity for detecting a small population of EVs being taken up by cells, but it is not possible to assess whether functional proteins are being transferred from the exosome into the cytoplasm of recipient cells in this experiment since only membranes were labeled. In this context, a recent review discussing the caveats of experiments designed to show that exosomes transfer information to recipient cells recommends using either fluorescent proteins targeted to membranes or EV transmembrane proteins fused to fluorescent tags to trace exosomal membranes [66] to avoid any possible influence of lipophilic dyes upon EV uptake [67,68], in combination with tagged soluble exosomal cargo proteins to follow proteins transferred into the cytoplasm. We tested the ability of EVs, produced from cells stably expressing a Tag-BFP reporter protein fused to the membrane anchoring region of K-Ras (HVR-CAAX), to be taken up into serum-starved cells treated exogenously with these vesicles and found that the majority of this reporter protein remained in the medium outside of cells (Fig 5), even after 96 hr of incubation.

Since several publications have reported the co-localization of EVs with lysosomal markers in recipient cells, we considered the possibility that any EVs taken up by cells might be targets for endolysosomal degradation [43,69,70]. However, treating cells with the v-ATPase inhibitor bafilomycin A1 (Fig 3) for 1 hr prior and throughout the co-culture with EVs, failed to significantly increase the efficiency of EV uptake as measured by a fluorescent reporter protein. While it remains possible that endolysosomal degradation is a factor in reducing our ability to record EV protein delivery into the cytoplasm in uptake assays, this experiment failed to support this hypothesis.

Our failure to detect transfer of functional K-Ras protein from cell-to-cell by EVs (or by any other mechanism) suggests that transfer may be a rare event. A recent report of EV-to-cell transfer using a Cre-loxP system to investigate Cre mRNA transfer from EVs-to-cells supports this contention [71]. In this study, when a Cre-expressing cell line was co-cultured for 1 week at a 1:1 ratio with an RFP-to-GFP Cre-regulated reporter line, no more than 1% of the reporter cells underwent eGFP^+^ conversion. *In vivo* analysis of EV uptake by injecting EVs from a Cre^+^ line (pre-treated with RNase and proteinase K to remove free RNA and protein) into reporter^+^ tumors yielded an even lower percentage of eGFP+ conversion (less than 0.06% of observed cells). However, even though the authors performed a number of controls to emphasize the role of Cre mRNA and exclude the role of Cre protein in conferring these small effects, it is known that purified, wild-type Cre protein can cross cell membranes and induce recombination in reporter cells similar to those used in the EV study at rates of 1.5–10% in a concentration dependent manner (0.8–5.8 μM) [72].

While there are caveats for studies using lipophilic dye or Cre recombinase protein reporters to demonstrate uptake of EVs, we acknowledge that our studies employing fusion proteins to measure intercellular transfer of proteins could also affect the dynamics of EV release, delivery, and uptake. Although we have analyzed the size and morphology of exosomes from U87MG cells [1], we cannot be absolutely certain that U87MG cells stably expressing nLuc-HA-K-Ras fusion protein do not produce exosomes that differ in their size or morphology, although this seems unlikely Moreover, we did not test whether EVs released from U87MG cells stably expressing Tag-BFP-HVR or nLuc-HA-K-Ras are capable of improving cell viability similarly to endogenous EVs released from U87MG cells.

However, unlike experiments in which EVs are incubated with cells to study uptake, the co-culture BiLC assay we used to test for functional, intercellular transfer of K-Ras would in principle have detected functional K-Ras transfer from any size of EV. This system also mitigates concerns about incubating target cells with supraphysiological amounts of EVs, and it should also report on other non-EV modes for transferring K-Ras from cell-to-cell. Moreover, qualitative analysis of nLuc-HA-K-Ras (anti-HA), Flag-K-Ras (anti-Flag), and endogenous Ras (anti-pan-Ras) demonstrated that these are all primarily expressed in small EVs or exosomes (110,000*xg* pellets) with lower levels of expression in 16,500*xg* pellets (larger EVs and cellular debris) as observed in immunoblots from Fig 3D from this report and Fig 6A of our previous publication [1]. In addition, the values observed for the efficiency of incorporation of the larger fusion protein nLuc-HA-K-Ras into exosomes were within the range of values observed for the efficiency of the minimally-tagged Flag-K-Ras’ release to exosomes (Fig 3E). Nevertheless, although it seems unlikely, the fusion protein’s expression could alter the efficiency of exosome uptake and hence cell viability in a manner that inhibits the observed increase in cell viability. Another factor that could have contributed to our failure to detect transfer of K-Ras is that the threshold for detection of K-Ras-Raf RBD interaction by the BiLC assay is too high to be useful as a reporter for EV delivery of K-Ras. Only 0.08–0.10% of the total cellular K-Ras fusion protein was released into exosomes on average (Fig 3). With a probable percentage of EV uptake at less than 2% based on the analyses in Fig 5 (average uptake at 24 hr was 1.993%), transfer of total K-Ras to a recipient cell would be likely less than 0.002% of the level of K-Ras in the nLuc-HA-K-Ras-expressing cells that were the source of exosomes.

Given the low level of K-Ras that is released in exosomes, and the low percentages of uptake predicted by our data, it is not surprising that we failed to identify conditions that facilitate cellular uptake of EVs to observe BiLC in this assay. We incubated U87MG cells with exogenous EVs using a variety of conditions, some based upon prior publications, to test a number of conditions we hypothesized might enhance BiLC signals: 20–100 μg/ml small EVs, using a less selective EV preparation that should include small and large EVs, using epidermal growth factor (EGF) in combination with EVs [45], using Gas6 (an AXL receptor tyrosine kinase ligand) [73], and using Lipofectamine LTX in conjunction with a GALA peptide that putatively mimics viral fusogenesis [74]. Nevertheless, these experiments failed to produce a luminescent signal.

Prior work characterizing the effects of oncogenic Ras-bearing EVs on recipient cells observed these effects specifically in rat cells (RIE-1 or RAT-1) using soft agar assays [52,63]. In the Lee *et al*. study, the authors also examined the transfer of the oncogenic H-*ras* DNA from EV-to-cell using RAT-1 rat fibroblasts and observed phenotypic effects only following the first passage of cells from transformed foci, not in later passages or through *in vivo* analyses of tumors in mice. In addition, this group found that primary human endothelial cells (HUVECs) deprived of growth factors exhibited greater cell viability in the presence of EVs carrying oncogenic Ras protein, at least for the first two weeks. These results suggest a transient response of cells to EV treatment, and we propose that this response might occur through EV-to-cell surface contact signaling rather than uptake of oncogenic Ras protein.

We are unable to provide a mechanism for the statistically significant yet modest increase in cell viability that we observed when serum-starved U87MG cells cultured in the absence of serum were treated with exosomes derived from U87MG cells. (Mean percentage of increase in cell viability was 9% using 2 μg/ml and 10% using 10 μg/ml of exogenous exosome treatment; significance was detected only after 7 iterations of the experiment (Fig 2). With the lowest concentration we used (2 μg/ml) in these experiments, which still revealed a significant enhancement of viability, exosomes from 880,000 U87MG cells on average, harvested over 48 hr, were added to the medium of a well containing 15,000 U87MG recipient cells (Fig 3), which represents a 60-fold excess. It should be noted, however, that these conditions seem unlikely to be physiologically relevant.

Results of a small-scale experiment (n = 3) suggest that Ras activity is not the primary factor driving increased cell viability in serum-starved cells treated with exogenous exosomes. We treated serum-starved U87MG cells with exogenous exosomes carrying either Flag-K-Ras G12D (constitutive) or Flag-K-Ras S17N (dominant negative), and, on average, exosomes carrying either K-Ras mutant displayed improved viability over a control. For the G12D fusion, an increase of 11% in cell viability was observed while S17N demonstrated an increase of 8.5% on average. However, exogenous exosome treatment with either mutant failed to display statistical significance after 3 iterations of the experiment, and the level of significance was clearly even smaller when comparing cells treated with exogenous exosomes carrying the constitutive G12D mutant to exosomes carrying the dominant negative S17N mutant.

It is possible that factors independent of Ras activity are affecting cell viability in this experiment. It could be that a combination of factors affects cell viability and that it might be difficult to dissect these factors using statistical testing if numerous proteins or pathways provide minor contributions to this modest phenotype. The conditioned media is highly concentrated to prepare exosomes for these experiments; thus, exosome preparation might concentrate other media components that artifactually improve cell viability during serum starvation. Overall, due to a failure to observe a significant and quantifiable role for Ras activity in conferring this phenotype, and a potential that the phenotype might result from experimental artifact, we discontinued our pursuit of a mechanism for this effect.

Taken together, our studies suggest that released EVs might function outside of cells rather than be taken up by neighboring cells. Two recent studies propose possible functions for exosomes within the extracellular matrix (ECM) as opposed to within the cytoplasm of exosome-accepting cells. First, exosomal leukotriene B_4_ (LTB_4_) and LTB_4_ synthesizing enzymes were shown to be released with exosomes from neutrophils, and this group suggested that exosomal LTB_4_ sensitizes neutrophils towards a primary chemoattractant and participates in neutrophil recruitment to sites of inflammation in a manner dependent upon the LTB_4_ receptor on neutrophils [75]. Second, another group demonstrated that endolysosomal cell mutants inhibited at various steps required for exosome release still demonstrated defects in fibrosarcoma cellular migration [76]. In this study, exosomes could rescue cell motility defects in a manner dependent upon the amount of exosomal fibronectin, sorted to the exosome surface in an integrin binding-dependent manner. In a more recent study, comparisons of purified fibronectin to fibronectin-depleted exosomes implicate a role for exosomal fibronectin in regulating the speed, rather than the directionality, of migration [77]. While the detailed mechanisms for these groups’ findings are unclear and do not include evidence that an exosome-to-cell surface interaction induces specific effects, both studies found a requirement for factors upstream of exosome release in regulating cell motility. Future studies of specific exosome or EV-to-cell surface interactions that regulate extracellular signaling could clarify these mechanisms and inform the design of therapeutics for inflammation and/or tumor signaling pathways.

It is also possible that the primary purpose of EVs is to dispose of unwanted cellular constituents. In a recent review of EVs and cancer, the authors discussed this possible fate for EVs and also considered signaling and information transfer as other possible fates using the current literature to assess the level of support for these different functions [66]. Besides evidence that heparin sulfate proteoglycans act as receptors for EV internalization [78], the authors noted a scarcity of evidence to support a membrane fusion step that would be required for the transfer of soluble factors from EVs to the cytoplasm of neighboring cells. By contrast, the authors presented published evidence that EVs might serve to discard toxic or unnecessary factors such as miRNAs in greater abundance than their mRNA targets [70] and tumor repressor miRNAs released from aggressive cancer cells [79]. Moreover, the observation that EVs taken up by neighboring cells colocalized with lysosomal markers [43,69,70] suggests a degradative fate for membrane and soluble EV factors taken up by cells rather than sustained signaling and information transfer.

Overall, we conclude that if transfer of intra-exosomal proteins into recipient cells occurs, it must happen at a very low efficiency, and that probably insufficient K-Ras molecules could be transferred to directly initiate downstream ERK MAP kinase pathway signaling in the recipient cell. Our studies were unable to detect transfer of Ras protein from cell-to-cell, and reporter protein analysis of EV-to-cell transfer demonstrated that the majority of the EV reporter protein remained outside of cells, consistent with an extracellular role for exosomes in regulating cellular functions. Future research in this area should aim to increase the sensitivity of detection of protein cargo transfer between the EV and the target cell to validate specific intercellular signaling mechanisms, and should design experiments that better model the physiological environment.

## Supporting information

S1 Fig Dye-labeled exosomes transmit fluorescence to the cellular cytoplasm. Additional nuclei surface maps overlaid on DiD signal to support information from Fig 1C(A) and 1D(B).
(TIF)

S2 Fig Supporting information for Fig 2B. Graph prepared using GraphPad Prism as detailed for Fig 2C.
(TIF)

S3 Fig Supporting information for Fig 3. (A) Leftmost blot from Fig 3B and 3C (those labeled with CE), uncropped here. Blotted with primary and secondary antibodies indicated, using 2 sequential steps to probe the fusion protein of interest first and then probe tubulin for normalization. (B) Rightmost blot from Fig 3B and 3C (those labeled with CC), uncropped here. Blotted with primary and secondary antibodies indicated, using 2 sequential steps to probe the fusion protein of interest first and then probe tubulin for normalization. (C) Separate channels represented in Fig 3D. C) Separate channels represented for green (680) and red (800) signals observed in Fig 3D for anti-HA and anti-Ras detection, respectively, to observe expression of nLuc-HA-K-Ras. The anti-integrin α5 blotted section was cut from the gel prior to transfer to analyze expression in the same lanes.CE = both BiLC fusions (nLuc-HA-K-Ras and cLuc-Flag-Raf-RBD) were stably coexpressed in a single line, CC = BiLC fusions were co-cultured together stably expressed in separate cell lines. WT, G12D, Y40C indicate which form of the nLuc-HA-K-Ras fusion protein is stably expressed in U87MG.
(TIF)

S1 Table (XLSX)

S2 Table (XLSX)

S3 Table (XLSX)
