# Supplementary figures and images for: Failure to detect functional transfer of active K-Ras protein from extracellular vesicles into recipient cells in culture
Source: PLoS One. 2018 Sep 7;13(9):e0203290. doi: 10.1371/journal.pone.0203290 (PMC6128481; doi:10.1371/journal.pone.0203290)

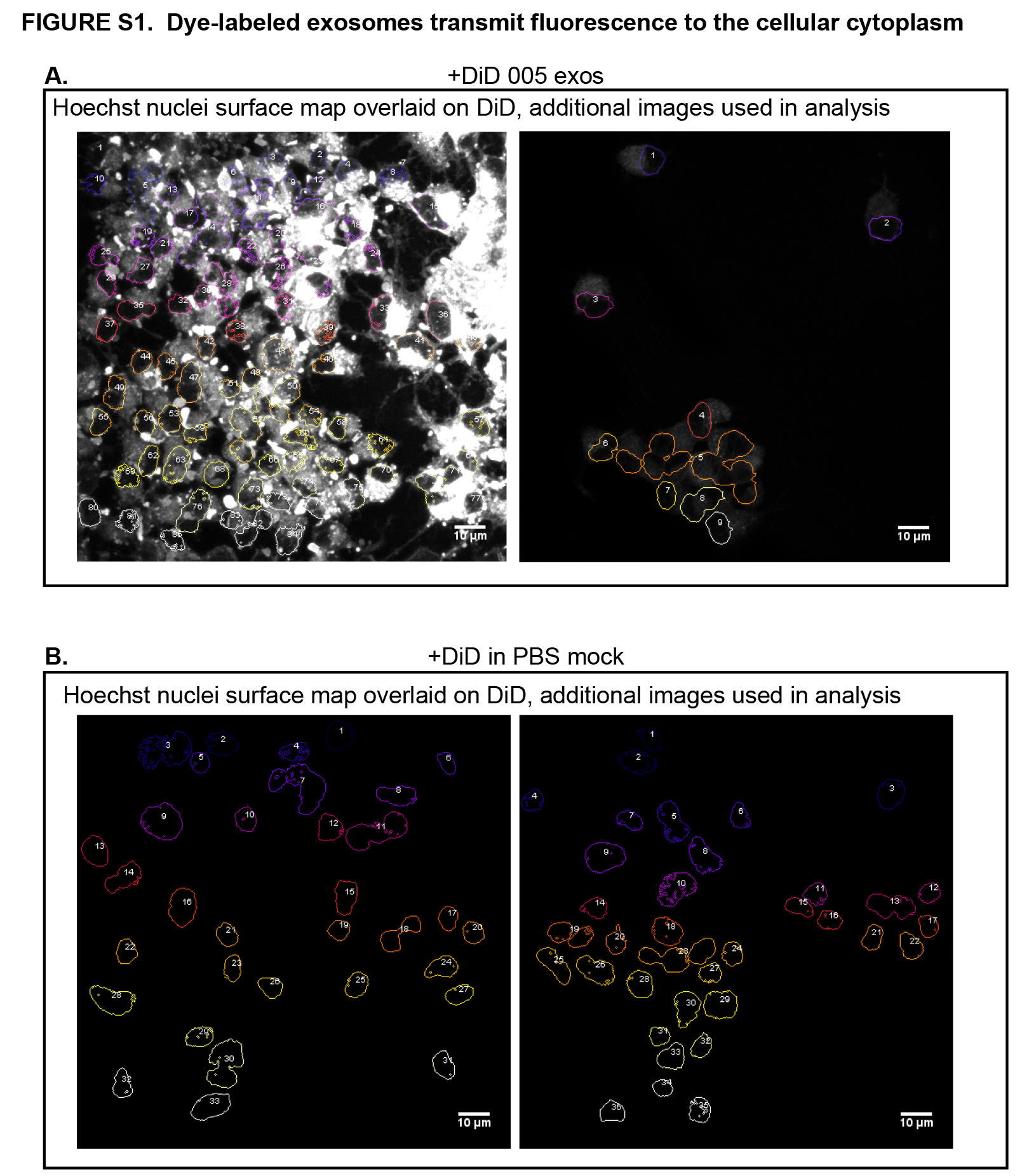

Supplement: S1 Fig — Additional nuclei surface maps overlaid on DiD signal to support information from Fig 1C(A) and 1D(B). (TIF) [file pone.0203290.s001.tif]

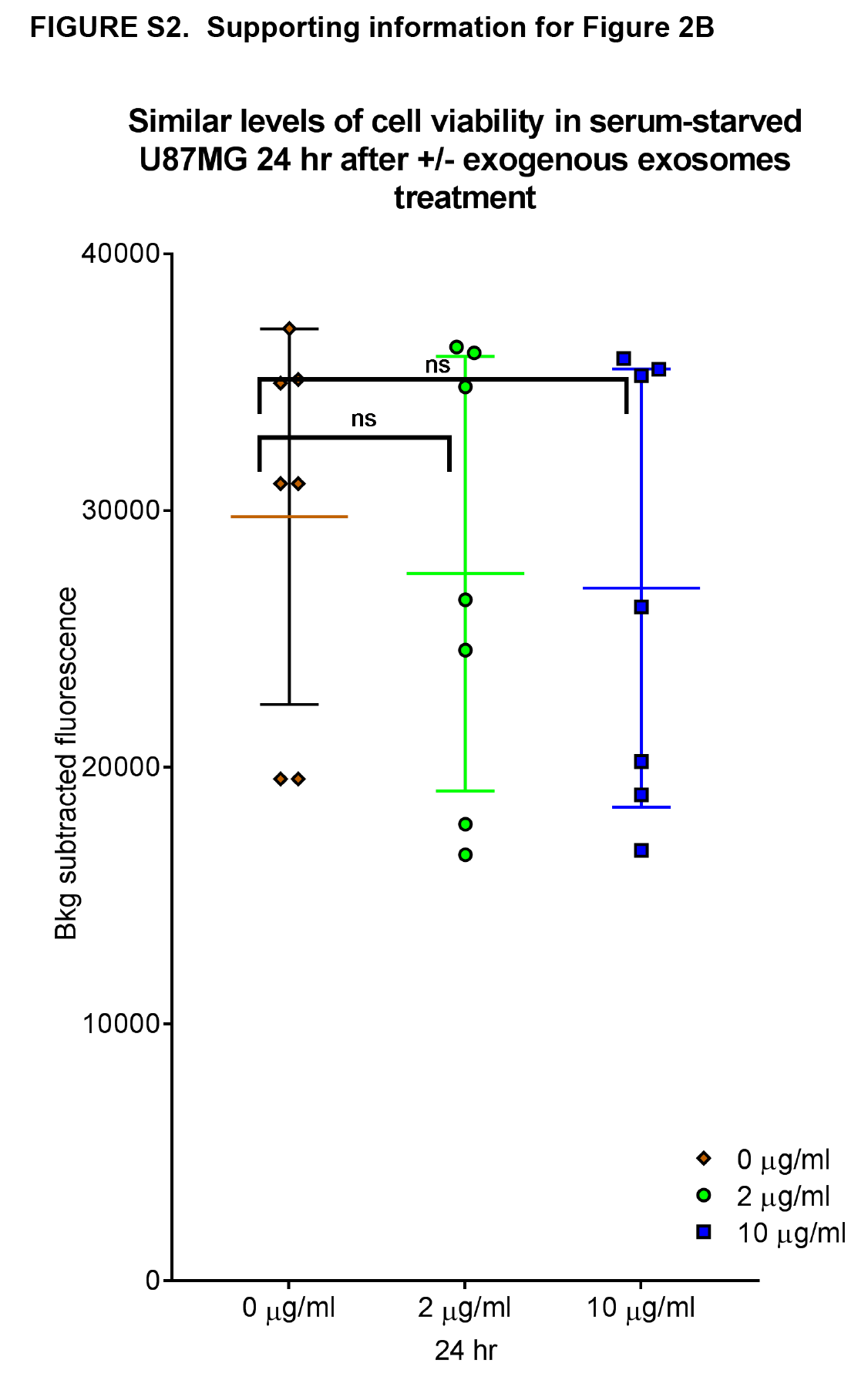

Supplement: S2 Fig — Graph prepared using GraphPad Prism as detailed for Fig 2C. (TIF) [file pone.0203290.s002.tif]

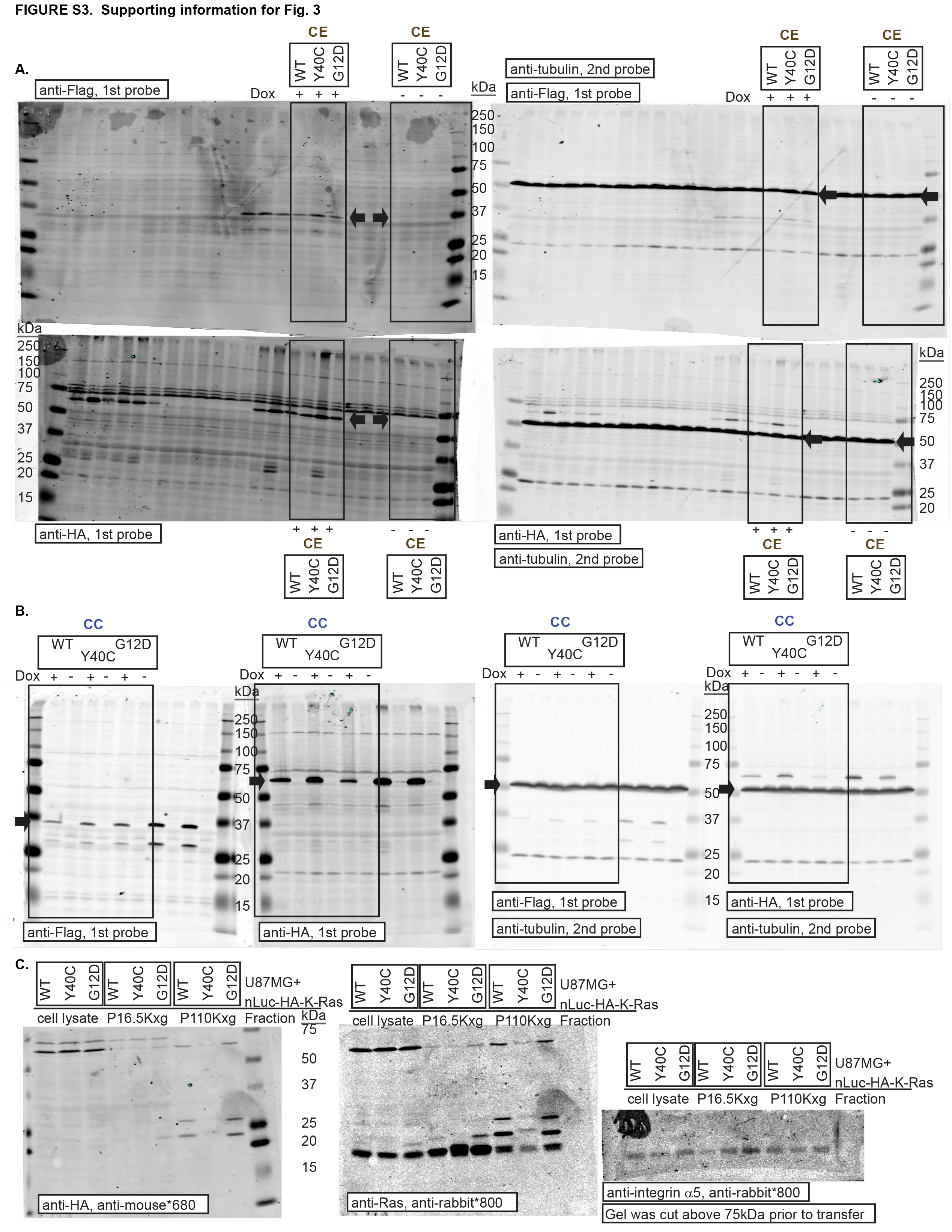

Supplement: S3 Fig — (A) Leftmost blot from Fig 3B and 3C (those labeled with CE), uncropped here. Blotted with primary and secondary antibodies indicated, using 2 sequential steps to probe the fusion protein of interest first and then probe tubulin for normalization. (B) Rightmost blot from Fig 3B and 3C (those labeled with CC), uncropped here. Blotted with primary and secondary antibodies indicated, using 2 sequential steps to probe the fusion protein of interest first and then probe tubulin for normalization. (C) Separate channels represented in Fig 3D. C) Separate channels represented for green (680) and red (800) signals observed in Fig 3D for anti-HA and anti-Ras detection, respectively, to observe expression of nLuc-HA-K-Ras. The anti-integrin α5 blotted section was cut from the gel prior to transfer to analyze expression in the same lanes.CE = both BiLC fusions (nLuc-HA-K-Ras and cLuc-Flag-Raf-RBD) were stably coexpressed in a single line, CC = BiLC fusions were co-cultured together stably expressed in separate cell lines. WT, G12D, Y40C indicate which form of the nLuc-HA-K-Ras fusion protein is stably expressed in U87MG. (TIF) [file pone.0203290.s003.tif]
